# Fetus-in-Fetu: A PRISMA-Informed Narrative Review With an Illustrative Prenatal Case

**DOI:** 10.14740/jocmr6423

**Published:** 2026-06-30

**Authors:** Agata Leszczynska-Wierzba, Michal Buczynski, Aleksandra Jasinska, Katarzyna Luterek, Denis Aduku, Aleksandra Slowakiewicz, Katarzyna Balcerak, Katarzyna Zapalowicz, Dariusz Madajczak, Beata Borek-Dzieciol, Bozena Kociszewska-Najman, Arkadiusz Krzyzanowski, Przemyslaw Kosinski, Wiku Andonotopo, Muhammad Adrianes Bachnas

**Affiliations:** a Department of Obstetrics, Perinatology, Gynecology and Reproductive Medicine, Medical University of Warsaw, Warsaw, Poland; b Department of Cardiac and General Pediatric Surgery, Medical University of Warsaw, Warsaw, Poland; c Department of General Pediatric Surgery and Urology, Medical University of Warsaw, Warsaw, Poland; d 1st Department of Obstetrics and Gynecology, Medical University of Warsaw, Warsaw, Poland; e Department of Neonatology and Rare Diseases, Medical University of Warsaw, Warsaw, Poland; f Department of Obstetrics and Pathology of Pregnancy, Medical University of Lublin, Lublin, Poland; g Department of Obstetrics and Gynecology, Fetomaternal Division, Women Health Center, Ekahospital BSD City, Serpong, Tangerang, Banten, Indonesia; h Faculty of Medicine, Maranatha Christian University, Bandung, West Java, Indonesia; i Department of Obstetrics and Gynecology, Fetomaternal Division, Medical Faculty of Sebelas Maret University, Dr. Moewardi Hospital, Solo, Surakarta, Indonesia

**Keywords:** Fetus-in-fetu, Teratoma, Embryology, Congenital abnormalities, Prenatal diagnosis, Neonatal surgery, Parasitic twin

## Abstract

Fetus-in-fetu (FIF) is an uncommon congenital anomaly arising from aberrant monozygotic twinning, in which a malformed parasitic twin becomes incorporated within the body of the host. Despite its rarity, FIF remains clinically relevant because it is frequently misdiagnosed as a teratoma, particularly when detected outside the neonatal period or at atypical anatomical sites. This review present a Preferred Reporting Items for Systematic Reviews and Meta-Analyses (PRISMA)-informed narrative synthesis of the human literature on FIF, integrating embryological concepts, diagnostic imaging features, genetic observations, and surgical management. A systematic search of major electronic databases identified 45 publications meeting predefined eligibility criteria. From these, 25 core studies were selected for in-depth narrative analysis based on diagnostic confirmation, methodological clarity, and avoidance of overlapping case reports. To contextualize the literature, a previously unreported human case of FIF associated with omphalocele and complex congenital cardiac anomalies was also described, illustrating current prenatal diagnostic pathways and multidisciplinary postnatal care. Across reported cases, FIF is most often diagnosed in infancy and is typically benign following complete surgical excision. Distinguishing FIF from mature teratoma relies on recognition of organized axial structures, symmetry, and shared monozygotic genetic identity. Although malignant transformation is rare, incomplete resection and immature tissue components warrant long-term surveillance. Improved prenatal imaging has enhanced early recognition of FIF, yet its developmental mechanisms remain incompletely understood. Future progress will likely depend on coordinated clinical reporting and translational research addressing early embryonic asymmetry, diagnostic refinement, and evidence-based follow-up strategies.

## Introduction

Fetus-in-fetu (FIF) is a rare congenital anomaly in which a malformed parasitic twin is found within the body of its host, arising from aberrant monozygotic twinning. Since its earliest clinical descriptions, FIF has drawn sustained attention from embryologists, pediatric surgeons, radiologists, and pathologists because it challenges conventional distinctions between developmental malformation and neoplastic growth [1, 2]. The term was introduced in the early 19th century by Johann Friedrich Meckel and later refined by Lewis, who emphasized the presence of organized axial development as a defining feature separating FIF from teratomas [3]. Despite more than a century of discussion, FIF remains incompletely understood and continues to pose diagnostic and conceptual difficulties.

From an embryological perspective, FIF is thought to result from abnormal division of a monozygotic embryo, most likely during early post-implantation stages. In this scenario, one twin becomes incorporated into the developing body of the other and survives through vascular support from the host. The parasitic twin typically demonstrates varying degrees of structural organization, including vertebral elements, limb buds, and rudimentary organ primordia, reflecting arrested but patterned development [4]. These features are central to the distinction between FIF and mature teratomas, which originate from totipotent germ cells and lack a coherent body plan.

The boundary between FIF and highly organized, or fetiform, teratomas remains a subject of debate. Some authors have proposed that FIF represents the extreme end of a teratoma spectrum, characterized by advanced differentiation rather than true twinning [5]. Others argue that consistent findings of axial symmetry, organoid arrangement, and monozygotic genetic identity support classification as a developmental anomaly rather than a neoplasm [6]. This distinction is not merely semantic, as it influences diagnostic interpretation, surgical planning, postoperative surveillance, and counseling of affected families.

Epidemiologically, FIF is extremely uncommon, with an estimated incidence of approximately one case per 500,000 live births and a reported male predominance [2]. Most cases are identified in infancy, often following evaluation of an abdominal or retroperitoneal mass. Nonetheless, delayed diagnosis into adolescence or adulthood has been reported, frequently with initial misclassification as a teratoma or other intra-abdominal tumor [7]. Although the retroperitoneum is the most frequently reported location, FIF has been described in a wide range of anatomical sites, including the cranial cavity, mediastinum, sacrococcygeal region, oral cavity, and scrotum, underscoring its anatomical variability [8–10].

Advances in prenatal ultrasonography, cross-sectional imaging, and postnatal surgical techniques have improved recognition and management of FIF. However, the rarity of the condition limits opportunities for systematic study, and much of the literature consists of isolated case reports or small series. As a result, uncertainties persist regarding embryological mechanisms, optimal diagnostic pathways, and long-term outcomes.

The present work aims to synthesize available clinical and biological knowledge on FIF through a narrative review informed by a systematic literature search. In addition to summarizing embryological concepts, imaging characteristics, genetic findings, and surgical management strategies, we present a rare human case of FIF associated with omphalocele and complex congenital heart disease. By integrating this case within the broader literature, this review seeks to provide a balanced and clinically relevant overview of FIF while highlighting areas where further collaborative research is needed.

## Methodology

### Study design and reporting framework

This work was designed as a narrative review informed by a systematic literature search, with reporting guided by the Preferred Reporting Items for Systematic Reviews and Meta-Analyses (PRISMA) 2020 framework. PRISMA principles were applied to structure the processes of study identification, screening, and eligibility assessment, while allowing interpretive flexibility appropriate for a rare condition predominantly documented through individual case reports and small case series. Because the aim was synthesis and critical interpretation rather than exhaustive aggregation, this review was not registered in PROSPERO. Accordingly, this work should be interpreted as a PRISMA-informed narrative review rather than a comprehensive systematic review, reflecting both the rarity of FIF and the predominance of descriptive case-based evidence in the literature. No protocol amendments were introduced after study initiation.

### Information sources and search strategy

A systematic search was conducted across PubMed/MEDLINE, EMBASE, Scopus, and Google Scholar. The search covered publications from January 1960 to October 2024, reflecting the historical span of FIF reporting. The literature search was intentionally closed in October 2024 to allow sufficient time for study screening, synthesis, and manuscript preparation, acknowledging that isolated case reports published thereafter are unlikely to alter the conceptual or clinical conclusions of this review. Search terms incorporated both classical and contemporary nomenclature, including FIF, parasitic twin, fetiform teratoma, monozygotic twinning anomaly, intrauterine fetal mass, and neonatal abdominal tumor. Boolean operators were used to balance sensitivity and specificity. Reference lists of relevant articles were manually screened to identify additional publications. Duplicate records were removed using database tools and manual verification.

### Eligibility criteria and study selection

Eligible studies reported human cases of FIF with clinical, surgical, imaging, or pathological confirmation sufficient to distinguish the condition from teratoma or related entities. Only full-text articles published in English were considered. Exclusion criteria included non-human studies, purely theoretical reports, incomplete case descriptions, and publications lacking adequate diagnostic detail. Titles and abstracts were screened independently by two reviewers (PK and WA), with disagreements resolved by discussion and consensus.

**Figure 1:**
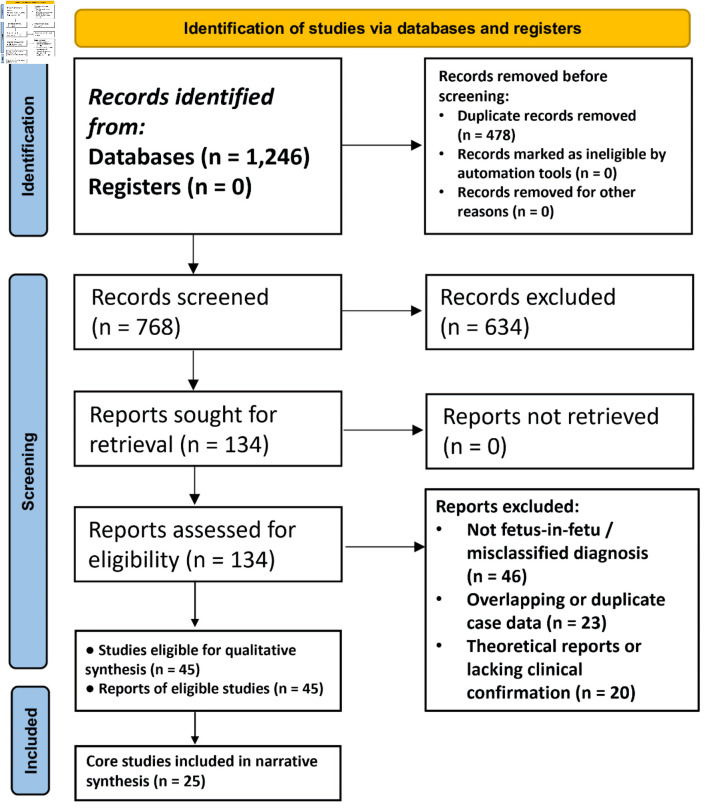
PRISMA 2020 flow diagram of study identification, screening, eligibility, and inclusion. This PRISMA 2020 flow diagram summarizes the identification and selection of studies evaluating fetus-in-fetu (FIF). A total of 1,246 records were identified through database searching (PubMed, EMBASE, Scopus, and Google Scholar). After removal of duplicate records, 768 unique records underwent title and abstract screening, of which 634 were excluded for irrelevance or misclassification. Full-text assessment was performed for 134 reports, with 89 excluded due to absence of confirmed FIF diagnosis, overlapping or duplicate case data, or insufficient clinical confirmation. In total, 45 studies met eligibility criteria for qualitative synthesis. From these, a core subset of 25 studies was selected for focused narrative synthesis and detailed citation in this review, based on predefined criteria emphasizing diagnostic certainty, clinical relevance, methodological completeness, and avoidance of overlapping case reporting. This two-stage inclusion approach aligns with PRISMA principles while allowing transparent and focused synthesis in the context of a rare condition with heterogeneous reporting. PRISMA: Preferred Reporting Items for Systematic Reviews and Meta-Analyses.

The search yielded 1,246 records. After removal of duplicates, 768 unique articles were screened, and 134 underwent full-text review. Forty-five publications met predefined eligibility criteria. As this review was narrative rather than comprehensive, a core set of 25 studies was purposefully selected from the eligible pool for detailed citation and synthesis. Selection was guided by diagnostic certainty, completeness of clinical and imaging data, historical or conceptual relevance, and avoidance of overlapping or duplicate case reporting. The study selection process is summarized in Figure 1, in accordance with PRISMA 2020 standards.

### Data extraction and synthesis

From the core studies, data were extracted on patient demographics, anatomical location of the parasitic twin, diagnostic timing and modality, operative findings, pathological features, embryological interpretations, and reported outcomes. Given the heterogeneity of study designs and the descriptive nature of the evidence base, findings were synthesized qualitatively. Results were organized thematically, encompassing epidemiological patterns, embryological concepts, imaging characteristics, genetic observations, surgical management, and ethical considerations relevant to clinical care.

### Risk of bias and certainty of evidence

Formal quantitative risk-of-bias assessment and meta-analysis were not undertaken, as the literature on FIF consists almost entirely of observational case reports and small series. Instead, methodological quality was appraised narratively, focusing on diagnostic verification, internal consistency, and completeness of reporting. The certainty of evidence was interpreted cautiously, acknowledging the inherent limitations of rare-disease literature.

### Ethical considerations

Ethical approval for inclusion of patient-specific clinical data and images was obtained in accordance with institutional requirements. Written informed consent was secured from the parents of the neonate for publication of prenatal imaging and surgical photographs. All identifying information was removed to protect patient confidentiality.

## Results and Findings

### PRISMA outcome narrative and evidence mapping

The systematic search identified 1,246 records across PubMed, EMBASE, Scopus, and Google Scholar. After removal of 478 duplicates, 768 unique titles and abstracts were screened. Of these, 634 were excluded due to irrelevance, lack of clinical correlation, non-human data, or misclassification. Full-text assessment was performed for 134 articles, resulting in the exclusion of 89 reports because of overlapping case descriptions, insufficient diagnostic confirmation, or absence of radiologic, surgical, or pathological substantiation. Forty-five studies fulfilled eligibility criteria for qualitative synthesis. From this group, a curated core of 25 studies was selected for detailed citation and analysis, representing the most methodologically complete, clinically informative, and historically influential contributions to the FIF literature. The study selection process is summarized in the PRISMA 2020 flow diagram (Fig. 1), and the characteristics of the included studies are detailed in Table 1 [1–25].

**Table 1:** Study Characteristics and Narrative Methodological Appraisal of Core Literature Included in the Review of Fetus-in-Fetu (FIF)

Author	Study type/design	Population/setting	Sample size	Method	Primary objective	Key findings	Limitations	Risk of bias
Lewis et al, 1961 [3]	Case report + analysis	Pediatric patient, UK	1	Clinical and pathology analysis	Differentiate FIF from teratoma	Introduced axial skeleton criterion	Pre-modern imaging	Moderate
Grant et al, 1969 [1]	Case report	Neonate, Australia	1	Surgical and pathological description	Describe clinical features of FIF	Early formal clinical characterization of FIF	Limited diagnostic tools	Moderate
Kakizoe et al, 1972 [23]	Case report	Neonate	1	Surgical findings	Describe scrotal FIF	Rare location	Single case	Moderate
Heifetz et al, 1988 [15]	Case series	Pediatric patients	5	Pathological analysis	Argue teratomatous origin	Challenged twin theory	Conceptual bias	Moderate
Eng et al, 1989 [4]	Case report	Infant, Taiwan	1	Imaging and histopathology	Correlate imaging with pathology	Demonstrated organized fetal structures	Single case	Moderate
Senyuz et al, 1992 [24]	Case report	Pediatric patient	1	Operative findings	Differentiate epignathus	Clarified oral cases	Single case	Moderate
Hing et al, 1993 [14]	Case report + genetics	Pediatric patient	1	STR analysis	Assess monozygotic origin	Genetic proof of twinning	Single case	Low–Moderate
Kim et al, 1993 [16]	Case report	Pediatric patient	1	Longitudinal imaging	Describe postnatal growth	Documented growth	Single case	Moderate
Chen et al, 1997 [10]	Case report	Prenatal diagnosis	1	Ultrasound, genetics	Assess prenatal diagnosis	Early prenatal confirmation	Limited follow-up	Moderate
Hanquinet et al, 1997 [13]	Case report	Pediatric patient	1	US and MRI	Compare FIF and teratoma	Imaging differentiation	Single case	Moderate
Sanal et al 1997 [25]	Case report	Neonate	1	Imaging and surgery	Report associated anomalies	Highlighted malformations	Single case	Moderate
Hopkins et al, 1997 [9]	Case report	Pediatric patient	1	Long-term follow-up	Report malignant recurrence	Demonstrated malignant potential	Exceptional case	Moderate
Fowler, 1998 [21]	Case report	Pediatric patient	1	Surgical exploration	Describe split notochord variant	Expanded spectrum	Atypical	Moderate
Thakral et al, 1998 [22]	Case report	Pediatric patient	1	Imaging and pathology	Review features	Confirmed markers	Narrative	Moderate
Hoeffel et al, 2000 [2]	Case series + review	Pediatric patients, France	87	Imaging and surgical review	Establish diagnostic criteria	Defined imaging and pathological hallmarks	Retrospective aggregation	Moderate
Shirani et al, 2005 [5]	Case report	Neonate	1	Operative and neurologic assessment	Describe parasitic twinning	Expanded anomaly spectrum	Not classic FIF	Moderate
Karaman et al, 2008 [7]	Case series	Pediatric patients	2	Surgical management	Report clinical presentation	Confirmed favorable outcomes	Small sample	Moderate
Tofigh et al, 2008 [8]	Case report	Pediatric patient	1	Imaging and surgery	Document rare presentation	Supported embryologic origin	Single case	Moderate
Khalifa et al, 2008 [17]	Case report	Pediatric patient	1	Clinical and surgery	Describe diagnostic process	Reinforced imaging diagnosis	Single case	Moderate
Gupta et al, 2010 [19]	Case report	Neonate	1	Clinical imaging	Document congenital anomaly	Supported definition	Single case	Moderate
Sharma et al, 2012 [18]	Case report	Pediatric patient	1	Imaging and surgery	Report rare anomaly	Typical presentation	Limited novelty	Moderate
Has et al, 2013 [12]	Case report	Prenatal setting	1	Prenatal ultrasound	Assess prenatal detection	Improved early diagnosis	Limited window	Moderate
Huang et al, 2013 [20]	Case report	Pediatric patient	1	Radiologic assessment	Report unusual presentation	Expanded phenotype	Single case	Moderate
Sitharama et al, 2017 [6]	Case report + review	Pediatric patient	1	Imaging and review	Review embryology and diagnosis	Clarified differential diagnosis	Narrative synthesis	Low–Moderate
Kumar et al, 2019 [11]	Case report	Adult patient	1	Imaging and surgery	Adult FIF presentation	Delayed diagnosis	Single case	Moderate

This table includes all 25 core studies selected for qualitative synthesis in this review. Owing to the rarity of fetus-in-fetu, the available evidence consists predominantly of single case reports and small case series. Risk of bias was assessed narratively based on diagnostic certainty, completeness of reporting, and internal consistency rather than formal scoring tools. No study was excluded solely on the basis of methodological limitations. STR: short tandem repeat.

### Epidemiology and global distribution

Across the included literature, FIF emerges as an extremely rare developmental anomaly, with an estimated incidence of approximately 1 in 500,000 live births, although the reported incidence is likely underestimated due to underdiagnosis and historical misclassification [2]. Most reported cases originate from Asia, Europe, and North America, reflecting differences in access to advanced imaging and pediatric surgical services rather than true geographic clustering. A male predominance is consistently observed, with ratios ranging from two to three males per female case [11]. FIF has been reported almost exclusively in the context of monozygotic, monochorionic twinning, supporting a narrow developmental window of origin. No consistent maternal, familial, or environmental risk factors were identified across studies, reinforcing the interpretation of FIF as a sporadic embryologic event rather than a heritable or exposure-related condition.

### Embryologic timing and developmental divergence

**Figure 2:**
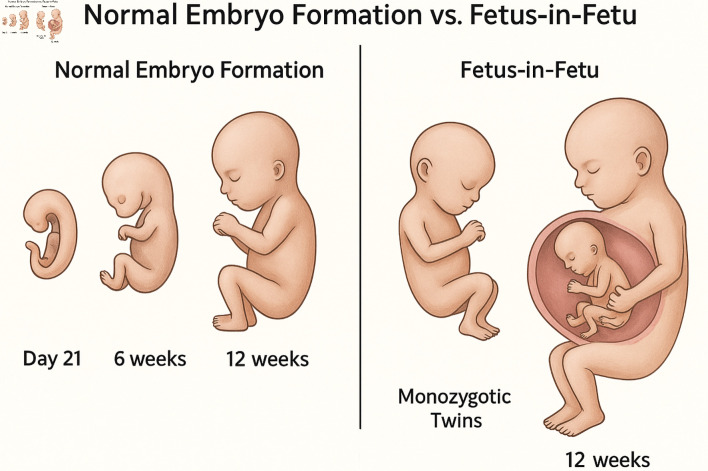
Schematic comparison of normal embryonic development and fetus-in-fetu (FIF) formation. This schematic illustrates key developmental differences between normal human embryogenesis and the proposed mechanism underlying FIF formation. In normal development, the zygote undergoes sequential cleavage, implantation, and organogenesis, resulting in a single, normally formed fetus. In the FIF pathway, incomplete separation of monozygotic twins during early post-implantation development leads to incorporation of one twin within the body of the host. The parasitic twin demonstrates arrested growth, remains dependent on the host’s circulation, and may develop rudimentary axial and limb structures. This figure is a conceptual representation synthesized from established embryological principles and clinical observations reported in the literature and is not derived from a single experimental or imaging dataset. Relative size and developmental timing are illustrative rather than to scale.

Analysis of embryologic data across the reviewed studies suggests that the divergence leading to FIF occurs during early post-implantation development, most plausibly between days 8 and 15 post-fertilization. This period coincides with splitting of the inner cell mass and early axial patterning. Table 2 summarizes the proposed embryological timeline, illustrating how asymmetric incorporation of one embryonic axis into the host embryo may occur. Figure 2 provides a conceptual contrast between normal embryonic development and the aberrant sequence that culminates in FIF formation. While precise mechanisms remain speculative, the recurring presence of vertebral segmentation and organized limb structures in FIF supports an origin after initiation of the primitive streak rather than a neoplastic process.

**Table 2:** Embryological Timeline and Proposed Developmental Disruptions Associated With Fetus-in-Fetu (FIF)

Embryonic stage (day/post-fertilization)	Developmental event	Potential disruption leading to FIF
Day 1–3	Fertilization and initial cleavages of zygote	Not applicable; monozygotic twinning has not yet occurred
Day 4–8	Blastocyst formation and early monozygotic twinning (dichorionic diamniotic possible)	Too early for FIF formation; may lead to separate twins
Day 8–13	Monochorionic monoamniotic twin formation; splitting of inner cell mass	Inclusion of one twin into the body of the other due to abnormal implantation or folding
Day 13–15	Gastrulation and primitive streak formation	Asymmetric incorporation of one twin; axial development initiates inside host
Day 16–21	Neurulation and early organogenesis; somite formation	Formation of vertebral axis and limb buds in parasitic twin; early differentiation within host embryo
Day 22–28	Cardiac looping, gut tube formation, and neural crest migration	Limited or failed development of cardiovascular and nervous structures in parasitic twin
Week 5–8	Organogenesis continues; limb and external genitalia become visible	Differentiation halts due to vascular insufficiency; structural organs remain rudimentary
Week 9–12	Fetal period begins; rapid growth and functional maturation	Parasitic twin remains encapsulated; host circulation dominates; growth suppressed

This table presents a conceptual synthesis of embryological stages relevant to FIF, informed by recurring observations in the reviewed literature and established principles of human embryology. It does not represent direct data extraction from individual studies, as no single report captures the complete developmental sequence. The proposed disruptions reflect plausible mechanisms inferred from clinical, imaging, and pathological findings. This framework is intended to aid interpretation rather than to assert definitive causality.

### Imaging findings and diagnostic evolution

Advances in prenatal and postnatal imaging have substantially altered the diagnostic landscape of FIF. First-trimester ultrasound may reveal an encapsulated mass with echogenic components, although early findings are frequently misinterpreted as omphalocele or teratoma [10, 12]. Retrospective analyses of early gestational imaging reported in the literature indicate that subtle indicators of FIF may be present but overlooked, particularly when coexisting congenital anomalies obscure diagnostic features. Second-trimester ultrasonography, including three-dimensional (3D) techniques, has enhanced anatomical delineation in atypical presentations, such as craniofacial attachment. Postnatally, computed tomography (CT) and magnetic resonance imaging (MRI) remain central to diagnosis and operative planning. CT provides superior visualization of axial skeletal elements, while MRI offers detailed assessment of soft tissue organization and vascular relationships [8, 13]. 3D reconstructions derived from cross-sectional imaging are frequently described as valuable adjuncts for surgical planning in anatomically complex cases.

### Genetic evidence and molecular confirmation

**Figure 3:**
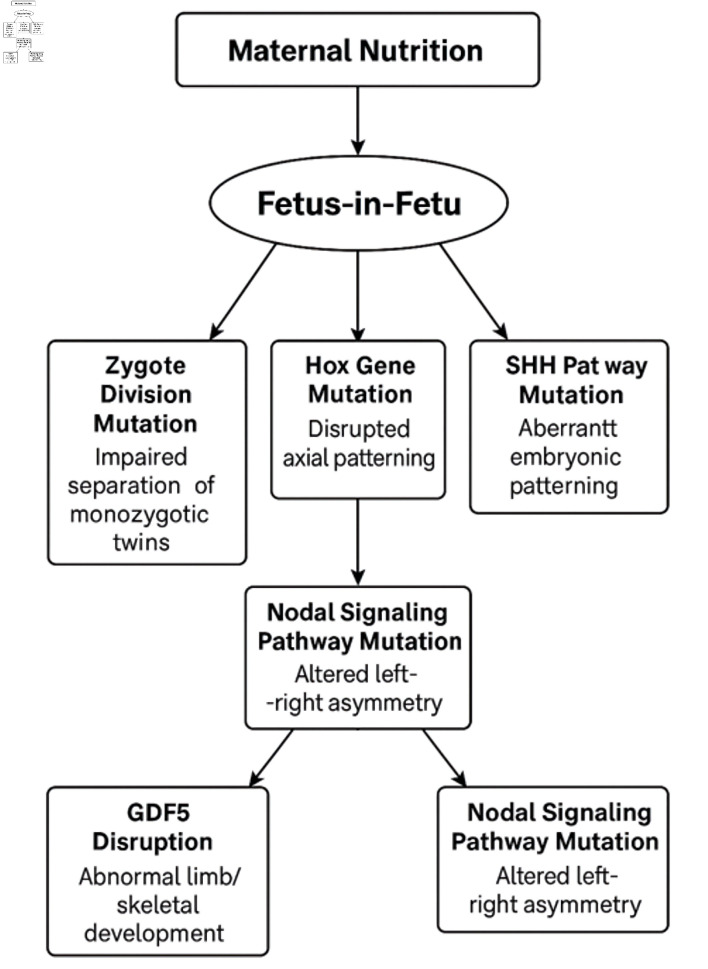
Conceptual genetic and developmental signaling framework proposed for fetus-in-fetu (FIF). This figure presents a conceptual framework summarizing developmental signaling pathways that have been hypothesized to contribute to the formation of FIF based on indirect evidence from embryology, monozygotic twinning biology, and reported genetic analyses in affected cases. The pathways shown, including disruptions in zygotic separation, axial patterning genes such as *Hox*, and key embryonic signaling cascades including Sonic Hedgehog (SHH) and Nodal, are not established causal mechanisms but represent biologically plausible contributors to asymmetric twin development. Maternal nutritional influences are illustrated as a modifying background factor rather than a direct etiologic driver, reflecting theoretical modulation of early embryonic signaling rather than proven causation.

Genetic analyses reported in the literature consistently demonstrate identical genetic profiles between the host and parasitic twin, most convincingly shown through short tandem repeat analysis and karyotyping [14]. These findings confirm a monozygotic origin and effectively exclude a neoplastic germ cell derivation. Across reported cases, FIF tissue typically exhibits a normal diploid karyotype without chromosomal instability, in contrast to teratomas. While no recurrent pathogenic mutations have been identified, the discordant morphogenesis between genetically identical twins suggests epigenetic dysregulation or asymmetric developmental signaling. Figure 3 synthesizes proposed genetic pathways involved in axial patterning and limb development, emphasizing their conceptual relevance rather than definitive causation.

### Environmental factors: evidence and limits

**Figure 4:**
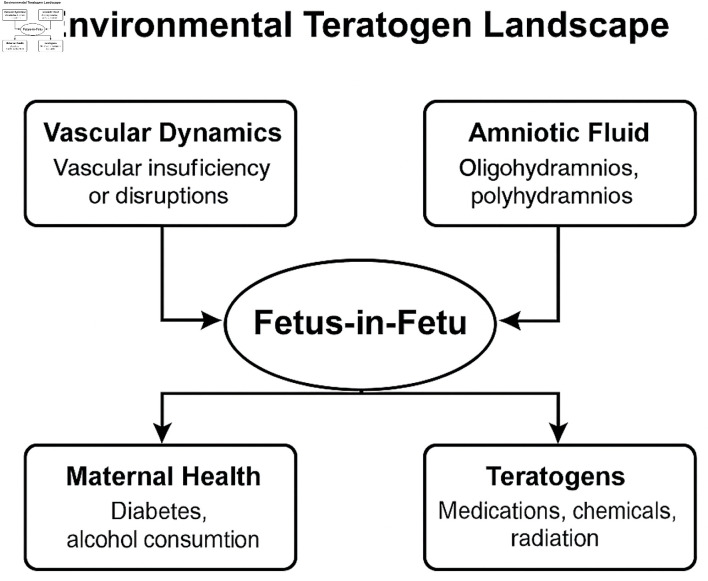
Conceptual overview of environmental and maternal factors discussed in relation to fetus-in-fetu (FIF). This figure depicts environmental and maternal factors that have been discussed in the literature as theoretical modifiers of early embryonic development in cases of FIF. The elements shown, including vascular dynamics, amniotic fluid abnormalities, maternal health conditions, and external teratogenic exposures, are not established causal risk factors but represent hypotheses proposed to contextualize asymmetric monozygotic twin development. The diagram is intended as a conceptual synthesis rather than evidence of direct etiologic relationships, reflecting the current absence of consistent epidemiologic or experimental data linking environmental exposures to FIF.

No study included in this review established a reproducible association between FIF and environmental exposures, maternal illness, infection, or teratogenic agents. Table 3 summarizes genetic and environmental factors discussed in the literature, highlighting the absence of empirical support for external triggers. Theoretical mechanisms such as vascular insufficiency or abnormal amniotic dynamics have been proposed but remain speculative. Figure 4 presents a conceptual overview of these hypotheses to contextualize ongoing debate rather than assert causality. Overall, the accumulated evidence strongly favors an intrinsic embryologic origin rooted in early monozygotic asymmetry.

**Table 3:** Genetic and Environmental Factors Implicated in the Development of Fetus-in-Fetu (FIF)

Category	Factor	Evidence	Implication
Genetic	Monozygotic twinning (monochorionic diamniotic origin)	STR analysis and karyotyping confirm shared genotype between host and parasitic twin	Strong support for included twin theory; not a neoplastic origin
Genetic	Normal diploid karyotype	Consistent findings in parasitic twin tissue across multiple studies	Helps distinguish FIF from teratomas, which may show aneuploidy or mosaicism
Genetic	No inherited mutation patterns	Lack of familial recurrence; no consistent mutation profile identified	FIF is likely a sporadic developmental error, not a heritable condition
Genetic	Epigenetic dysregulation	Hypothesized based on asymmetric differentiation despite identical genomes	Could explain why a parasitic twin fails to fully develop
Genetic	Zygote division mutation	Impairment in the zygotic division process leads to incomplete separation during monozygotic twinning	Results in the internalization of one twin and development of a parasitic fetus
Genetic	*Hox* gene mutation	Disruptions in *Hox* genes, which control body plan and axial patterning, may lead to abnormal segmentation of the parasitic twin	Disruption of normal body axis formation and incomplete development of the parasitic twin
Genetic	Sonic Hedgehog (SHH) pathway mutation	Disruptions in SHH signaling interfere with embryonic patterning, particularly limb and axial development	Results in asymmetric development and improper internalization of the parasitic twin
Genetic	Nodal signaling pathway mutation	Disruptions in Nodal signaling affect left-right patterning, potentially leading to abnormal asymmetry in twin positioning	Affects the positioning and development of the parasitic twin within the host
Genetic	Growth and differentiation factor 5 (GDF5) disruption	Disruptions in *GDF5*, involved in limb and skeletal patterning, may cause incomplete or arrested development of the parasitic twin	Leads to underdeveloped limbs or axial structures in the parasitic fetus
Environmental	Vascular supply disruption	Parasitic twin depends entirely on the host’s circulation; poorly perfused structures noted	May contribute to arrested organ development and asymmetry
Environmental	Abnormal amniotic cavity dynamics	Theoretical link to spatial entrapment of a parasitic twin within host embryo	Could play a role in internalization of twin during folding process
Environmental	Placental anastomoses	Shared vascular connections observed in some prenatal FIF diagnoses	May facilitate twin inclusion or maintain parasitic growth
Environmental	No clear association with teratogens or maternal illness	Epidemiologic data fail to support any consistent external trigger	Reinforces FIF as a non-environmentally induced anomaly

This table represents a conceptual synthesis of genetic and environmental factors discussed across the reviewed literature and informed by established embryological principles. The listed factors reflect proposed mechanisms inferred from clinical, imaging, pathological, and molecular observations rather than definitive causal relationships. No consistent environmental or inherited genetic triggers have been empirically confirmed for FIF. STR: short tandem repeat.

### Clinical presentation, management, and outcomes

**Figure 5:**
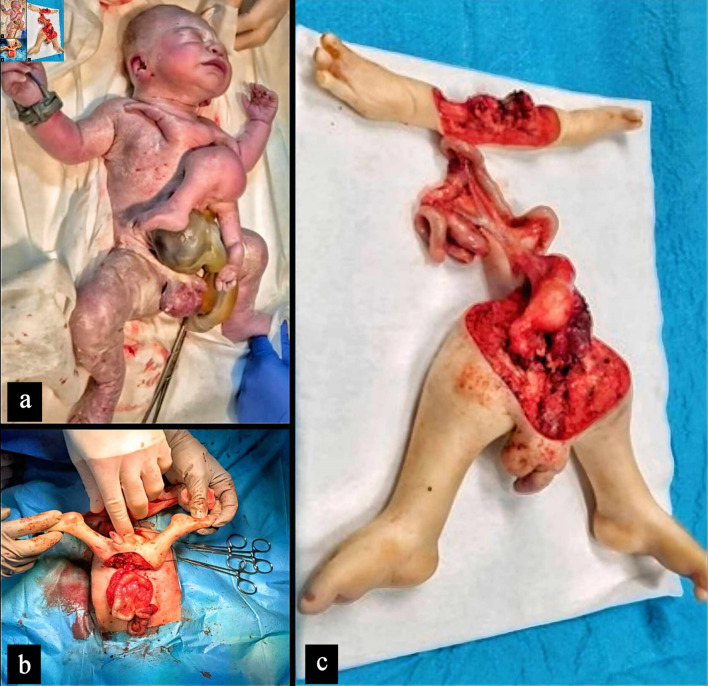
Clinical and operative documentation of fetus-in-fetu (FIF) in a neonate with associated omphalocele. (a) Full-term male neonate immediately after birth demonstrating a large omphalocele with an externally visible parasitic FIF attached cranial to the abdominal wall defect. (b) Intraoperative photograph obtained during staged surgical excision, illustrating organized anatomical structures of the parasitic twin and shared vascular attachments to the host. (c) Resected FIF specimen following complete removal, showing well-differentiated limbs, external genitalia, and identifiable visceral components. Histopathological examination confirmed the presence of mature cartilaginous tissue, neural elements, and gastrointestinal structures, supporting the diagnosis of FIF rather than teratoma. All images were obtained with parental informed consent and anonymized according to institutional ethical standards; scale is inferred from surgical context.

Clinically, FIF most often presents as a painless, enlarging abdominal mass in infancy, occasionally accompanied by feeding intolerance or gastrointestinal compression [2]. Rare adult presentations have been reported, typically discovered incidentally during evaluation for chronic abdominal symptoms [11]. Diagnostic differentiation from teratoma relies on the identification of axial skeletal elements, bilateral symmetry, and organized organ primordia. These distinguishing features are summarized in Table 4 and contrasted with teratomas and parasitic twins in Table 5. Surgical excision remains the definitive treatment, with excellent outcomes following complete resection. Although rare, malignant recurrence has been documented, underscoring the importance of thorough excision and postoperative surveillance [9]. Operative and pathological findings from a representative neonatal case are illustrated in Figure 5.

**Table 4:** Commonly Accepted Diagnostic Markers and Modalities Used in the Evaluation of Fetus-in-Fetu (FIF)

Category	Marker/feature	Modality	Diagnostic relevance
Imaging	Axial vertebral column	CT, MRI, prenatal ultrasound	Gold standard feature distinguishing FIF from teratoma; indicates embryonic origin
Imaging	Organized limb buds and symmetry	CT, MRI	Supports diagnosis of parasitic twin; shows structured development
Imaging	Encapsulated mass with cystic and solid components	Ultrasound, CT	Suggests inclusion phenomenon within host body; often seen in retroperitoneum
Genetic	Identical STR profile to host	Molecular genetics (PCR, STR analysis)	Confirms monozygotic origin; excludes neoplastic (teratomatous) origin
Genetic	Normal diploid karyotype	Karyotyping	Helps differentiate FIF from teratomas, which may show chromosomal abnormalities
Histopathology	Presence of vertebral elements and multi-organ tissues	Microscopic examination	Definitive confirmation; distinguishes from disorganized teratomatous growth
Tumor markers	Low or normal alpha-fetoprotein (AFP)	Serum analysis	Helps rule out yolk sac tumors or malignant germ cell tumors
Tumor markers	Negative β-hCG	Serum analysis	Further differentiates from certain teratomatous malignancies

This table summarizes diagnostic markers and investigative modalities that are consistently described across reported cases of FIF and widely used in clinical practice. The listed features reflect convergent imaging, genetic, and histopathological criteria rather than outcomes from comparative or prospective studies. Diagnostic relevance is based on cumulative clinical experience and repeated observations in the literature. STR: short tandem repeat; β-hCG: beta human chorionic gonadotropin; CT: computed tomography; MRI: magnetic resonance imaging; PCR: polymerase chain reaction.

**Table 5:** Conceptual Clinicopathological Comparison of Fetus-in-Fetu (FIF), Mature Teratoma, and Parasitic Twin

Feature	FIF	Mature teratoma	Parasitic twin
Presence of axial skeleton	Yes	No	Yes
Bilateral symmetry	Yes	No	Often
Encapsulated mass	Yes	Yes	No
Monozygotic origin	Yes	No	Yes
Organ primordia	Yes	No	Yes
Disorganized germ layers	No	Yes	No
Malignancy potential	Rare	Possible	No
External attachment	No	No	Yes
Shared vasculature with host	Rare	No	Yes
Functional limbs/organs	Rare	No	Yes

This table provides a conceptual comparison based on recurrent clinicopathological features described across reported cases and established diagnostic criteria. The distinctions shown reflect consensus patterns used in clinical practice rather than results from comparative or prospective studies. The table is intended to aid diagnostic reasoning and differential classification, not to imply absolute or universal boundaries between entities.

### Ethical dimensions and prenatal counseling

**Figure 6:**
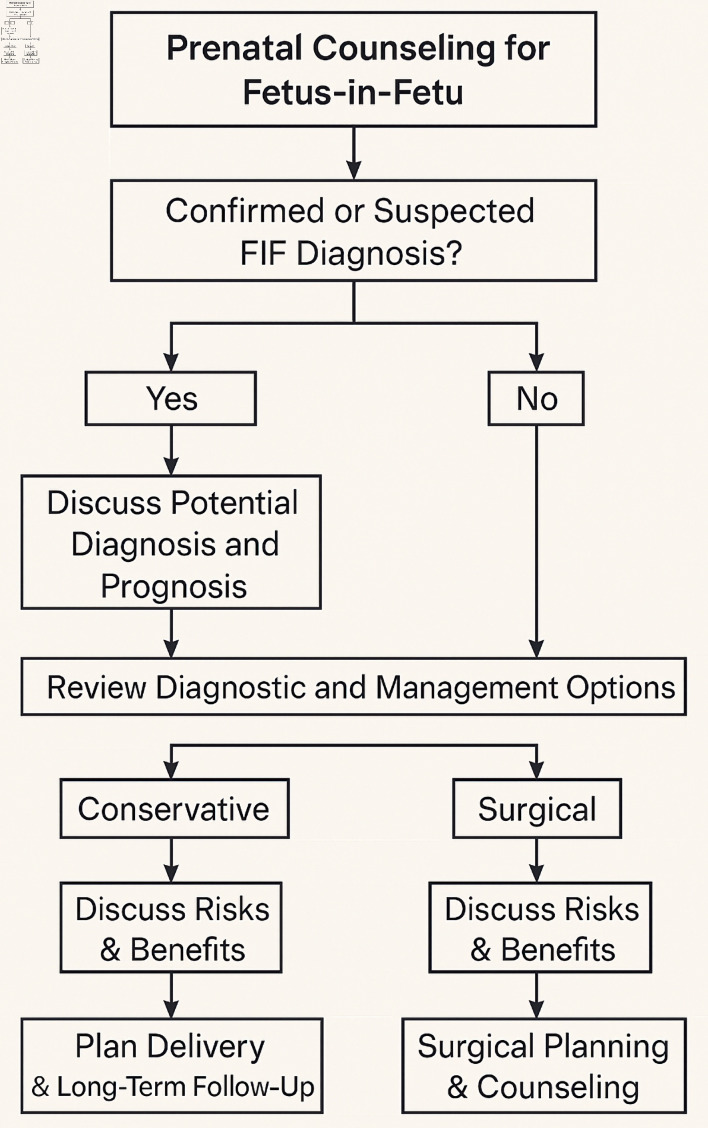
Conceptual prenatal counseling pathway for suspected or confirmed fetus-in-fetu (FIF). This schematic illustrates a structured, non-directive counseling pathway commonly used in clinical practice when FIF is suspected or confirmed prenatally. The diagram integrates diagnostic confirmation through imaging, anticipatory counseling regarding prognosis and potential complications, and discussion of conservative versus surgical management pathways, with decisions guided by gestational age, anatomical complexity, and maternal–fetal condition. The pathway represents a synthesized clinical framework rather than a formal guideline and is intended to support shared decision-making that incorporates medical, ethical, and cultural considerations.

Prenatal diagnosis of FIF introduces complex ethical and cultural considerations, particularly in regions where access to specialized care is limited or where cultural interpretations influence medical decision-making. Multidisciplinary counseling is essential to address parental concerns, balance surgical risks, and contextualize prognosis. Figure 6 presents a structured counseling framework integrating diagnostic certainty, anticipated clinical course, and ethical considerations. Across studies, empathetic communication and culturally sensitive engagement were central to achieving favorable outcomes and informed parental consent.

## Case Example

We present a rare and instructive case of FIF diagnosed prenatally and confirmed postnatally, notable for its coexistence with omphalocele and complex cardiac anomalies—an extremely uncommon constellation of findings in the literature. The diagnosis was first suspected during a first-trimester ultrasound scan at 12 weeks’ gestation, which revealed a massive omphalocele with associated cardiac malformation. Detailed imaging displayed multiple umbilical vessels and abnormal abdominal contents protruding from the fetal abdomen. A genetic workup, including microarray comparative genomic hybridization (CGH), revealed a normal chromosomal profile, thereby ruling out major aneuploidy.

**Figure 7:**
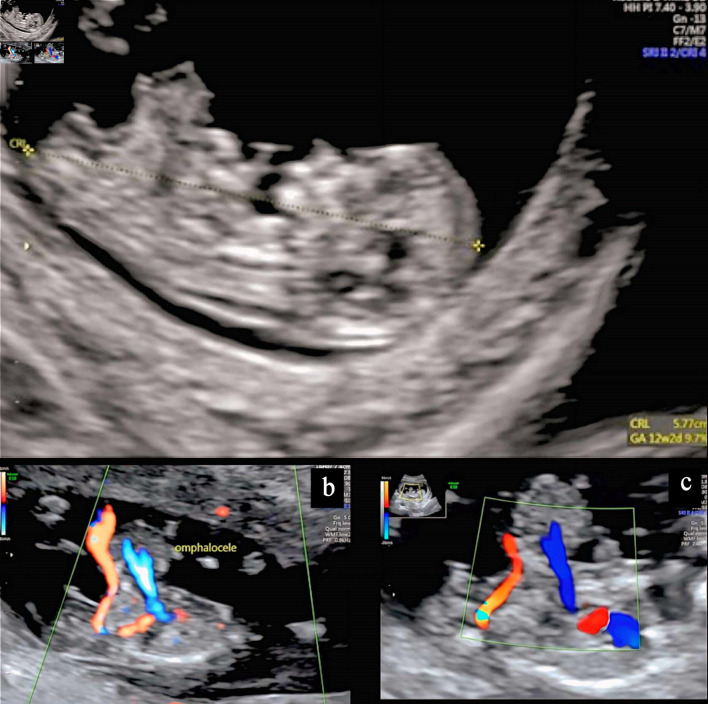
First-trimester ultrasonographic findings with retrospective indicators of fetus-in-fetu (FIF). (a) Standard two-dimensional ultrasound image obtained at 12 + 2 weeks of gestation demonstrates a structurally developed fetus with a large omphalocele and a suspected cardiac anomaly; at the time of examination, FIF was not suspected. (b) Color Doppler imaging shows umbilical vessels supplying the omphalocele. Immediately cranial to the defect, an irregular echogenic area is visible, which was retrospectively recognized as corresponding to the parasitic FIF. (c) Focused color Doppler view further delineates vascular flow within the omphalocele, while the adjacent region harboring the parasitic structure remains partially obscured by overlapping anatomy and complex associated anomalies. This figure illustrates the diagnostic challenges of early gestational imaging in FIF and highlights how subtle sonographic findings may only become apparent upon retrospective review. Relative scale is inferred from standard first-trimester ultrasound acquisition parameters.

Despite early sonographic findings, FIF was not suspected initially, primarily due to the dominant presence of omphalocele and the morphological ambiguity caused by the adjacent parasitic tissues. Figure 7 illustrates the prenatal ultrasound findings, highlighting how the parasitic twin was initially masked by overlapping anatomy and omphalocele. Mid-trimester anomaly scan confirmed the omphalocele containing both liver and bowel and established the cardiac diagnosis of double outlet right ventricle (DORV). The patient was referred to a tertiary center for advanced fetal care and delivery planning. A planned cesarean section was performed at 38 weeks and 4 days, delivering a male neonate weighing 3,150 g with umbilical cord pH 7.35. On postnatal examination, the omphalocele was clearly visible and revealed an unexpected parasitic twin structure. The parasitic entity included rudimentary upper and lower limbs, normal male external genitalia, and was anchored approximately 3 cm below the omphalocele margin.

**Figure 8:**
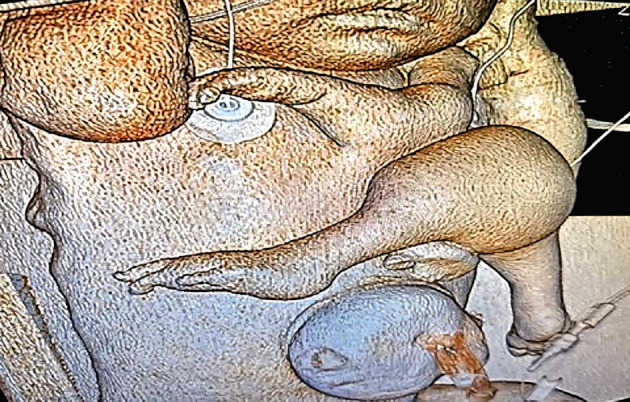
Preoperative three-dimensional computed tomography (CT) of fetus-in-fetu (FIF) with associated omphalocele. Three-dimensional reconstructed CT image of a neonate obtained shortly after birth demonstrates a large omphalocele arising from the lower abdominal wall. Immediately cranial to the defect, a well-formed lower limb belonging to a parasitic FIF is seen emerging from the anterior torso of the host. The parasitic limb exhibits clear skeletal alignment and surrounding soft tissue consistent with organized musculoskeletal development. This imaging modality was essential for delineating anatomical relationships between the parasitic structures, abdominal wall defect, and host viscera, and it informed staged surgical excision and abdominal wall reconstruction. Image orientation and relative scale are inferred from standard neonatal CT acquisition parameters.

Imaging, including CT with 3D rendering, delineated the structural components of the parasitic twin, confirming the presence of skeletal elements and partially organized viscera. The detailed anatomical relationships revealed in the preoperative imaging are illustrated in Figure 8, which was instrumental in planning the staged surgical approach. The diagnosis of FIF was established. Surgical management was performed in a staged manner. The first procedure involved resection of the parasitic twin, including pelvic and lower limb structures. The parasitic fetus contained a kidney, large and small intestines with blind ends, and was connected via a feeding vessel that emerged from the thoracic region of the host twin. Intraoperative and anatomical details of the parasitic twin are illustrated in Figure 5, showcasing the surgical findings and the extracted FIF specimen. The vessel was ligated, and the parasitic components were removed through an extended incision to access residual upper limb structures. Primary closure of the omphalocele was achieved. Intraoperatively, a shared connection involving the urachus of the parasitic twin and the umbilical cord of the host was identified—a unique anatomical feature not widely documented in prior FIF cases. Following resection, the neonate underwent aortic arch reconstruction for the hypoplastic segment and pulmonary artery banding, with delayed ventricular septal defect (VSD) repair planned for a future stage.

The neonate recovered well and was discharged home 4 weeks postoperatively in stable condition. Histopathological evaluation confirmed well-differentiated tissues, including cartilaginous, neural, and gastrointestinal elements, consistent with the diagnosis of FIF rather than teratoma. This case underscores the complexity and diagnostic challenge of FIF when coexisting with major congenital anomalies and highlights the critical role of multidisciplinary fetal-maternal care, surgical precision, and prenatal imaging in managing such rare entities. The presence of omphalocele and DORV likely masked the parasitic twin, delaying its recognition until postnatal inspection and imaging. To our knowledge, this is one of the very few reported cases of FIF with confirmed omphalocele and complex congenital heart disease, positioning it as a unique contribution to the global body of FIF literature.

## Discussion

### Revisiting FIF as a developmental entity

FIF occupies a distinctive position within human developmental pathology, situated at the intersection of monozygotic twinning, embryologic asymmetry, and surgical disease. From Meckel’s early descriptions to Lewis’s decisive separation of FIF from retroperitoneal teratoma [3], the literature has gradually converged on the view that FIF represents a developmental anomaly rather than a neoplastic process. What emerges from the present synthesis is not novelty of presentation, but consistency of structure. Across decades and geographic settings, FIF demonstrates a recurring pattern of organized axial development embedded within a host twin, a finding reflected across the studies summarized in Table 1 [1–25]. This reproducibility supports the interpretation of FIF as a definable developmental entity rather than a pathological outlier.

### Differential diagnosis and the persistence of diagnostic ambiguity

Despite advances in imaging and molecular diagnostics, distinguishing FIF from mature teratoma and parasitic twinning remains clinically challenging. Lewis’s axial skeleton criterion continues to serve as a practical diagnostic anchor [3], reinforced by later observations from Eng et al, demonstrating that vertebral segmentation, bilateral symmetry, and coherent organ primordia are uncommon in teratomas [4]. This distinction carries clinical weight. Teratomas are neoplastic, genetically unstable, and associated with malignant risk, whereas FIF exhibits monozygotic identity and developmental organization. Parasitic twins, by contrast, exist along a separate continuum, remaining externally attached and partially autonomous. These conceptual boundaries, summarized in Table 5, are most consequential during prenatal counseling and operative planning, where diagnostic imprecision can distort prognostic expectations and parental decision-making, as illustrated in Figure 6.

### Embryologic mechanisms: inclusion rather than neoplasia

The balance of evidence favors the included-twin hypothesis as the most coherent explanation for FIF. Genetic confirmation of monozygotic origin, most convincingly demonstrated by Hing et al through short tandem repeat analysis [14], effectively excludes a germ-cell neoplastic origin in classic cases. The alternative fetiform teratoma theory proposed by Heifetz et al [15] has historical relevance, but struggles to account for the consistent presence of axial organization and genomic stability observed across confirmed FIF cases. The embryologic timeline synthesized in Table 2 suggests that asymmetric incorporation during the period of primitive streak formation offers a biologically plausible mechanism. Figure 2 provides a conceptual visualization of how minor perturbations during early embryonic folding may result in profound downstream anatomical divergence.

### Imaging as a translational bridge between theory and practice

**Figure 9:**
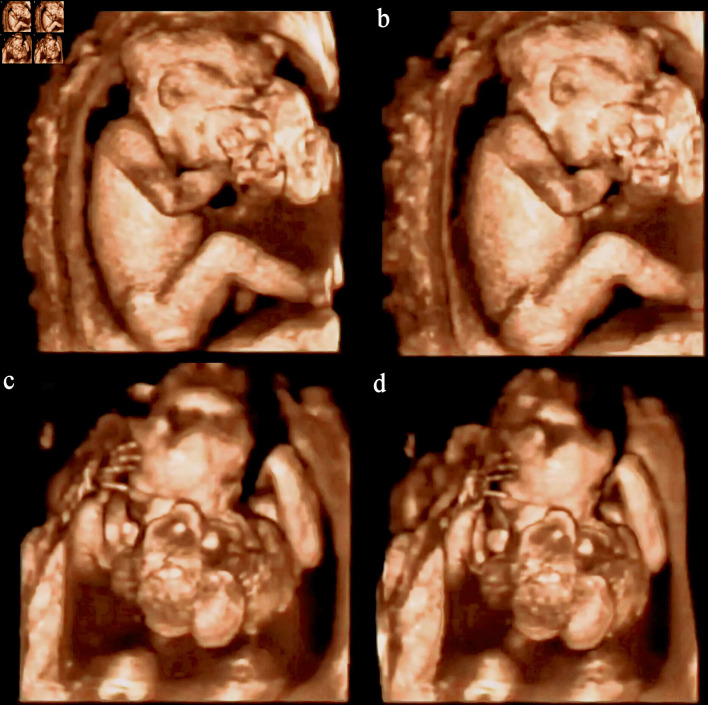
Second-trimester three-dimensional ultrasonography of a craniofacial fetus-in-fetu (FIF). (a, b) Lateral three-dimensional ultrasound views obtained during the second trimester demonstrate a fetus with a large, irregular mass arising from the facial region, initially raising suspicion for a craniofacial tumor. Detailed surface rendering reveals organized internal contours suggestive of segmented structures and rudimentary limb elements within the mass. (c, d) Frontal three-dimensional views further delineate a partially organized parasitic twin attached at the midface, with bulbous cranial components and incomplete bilateral symmetry. This case, derived from previously reported clinical imaging in the literature, illustrates a rare craniofacial presentation of FIF and contrasts with the primary case described in this review, which lacks second- and third-trimester imaging. The images emphasize the diagnostic value of mid-gestation three-dimensional ultrasonography in distinguishing FIF from craniofacial neoplasms and in facilitating anticipatory perinatal planning, particularly with respect to airway management. Relative scale is inferred from standard fetal biometric acquisition.

Imaging has transformed FIF from an incidental postoperative finding into a condition increasingly recognized along the prenatal–postnatal continuum. Early ultrasound detection remains difficult, particularly when FIF coexists with structural anomalies, yet prenatal reports by Chen et al and Has et al demonstrated that organized skeletal elements may be identifiable before birth [10, 12]. Retrospective analysis of early scans, as shown in Figure 7, underscores how diagnostic cues may be present but overlooked. Postnatally, CT and MRI provide complementary information, with CT delineating axial structures and MRI clarifying soft-tissue and vascular relationships [6, 13]. In anatomically complex cases, 3D reconstructions, exemplified in Figures 8 and 9, function not only as surgical tools but also as communicative aids during multidisciplinary planning.

### Clinical course, surgical strategy, and long-term risk

Most reported cases of FIF present in infancy, typically as a slowly enlarging abdominal mass, although rare adult presentations remind clinicians that diagnostic delay remains possible [11]. Surgical excision remains definitive, serving diagnostic, therapeutic, and psychological roles. FIF is usually benign following complete surgical excision; however, Hopkins et al’s report of malignant recurrence [9] and Kim et al’s documentation of postnatal growth [16] emphasize that complete excision and longitudinal follow-up are prudent. These observations suggest that risk is not intrinsic to FIF itself but is related to residual or immature tissue. Clinically, the challenge lies less in technical feasibility than in anticipating associated anomalies and coordinating care, as illustrated in the operative context shown in Figure 5.

### Environmental hypotheses and the limits of current evidence

A striking feature of the FIF literature is the absence of reproducible environmental or maternal risk factors. While hypotheses involving vascular disruption or amniotic dynamics have been proposed, none are supported by consistent empirical evidence. Table 3 and Figure 4 intentionally frame these mechanisms as conceptual models rather than established causative pathways. The sporadic occurrence of FIF, its monozygotic origin, and lack of familial recurrence collectively favor an intrinsic developmental asymmetry over environmental causation. In this respect, FIF highlights the limits of etiologic inference when studying rare embryologic events. Despite growing clinical recognition, FIF remains largely inaccessible to experimental interrogation, as no validated animal models currently reproduce the asymmetric monozygotic inclusion observed in humans. This absence of tractable models limits mechanistic insight and constrains predictive research, highlighting the need for closer collaboration between clinicians, developmental biologists, and imaging scientists. Emerging *in vitro* approaches to early human twinning and embryonic patterning may eventually offer partial analogues, but at present FIF knowledge continues to rely almost entirely on carefully documented human cases.

### Ethical and cultural contexts of care

FIF frequently extends beyond biological anomaly into ethical and cultural domains. In some settings, FIF may be interpreted through spiritual or symbolic frameworks, influencing parental acceptance of intervention [12]. The literature suggests that successful management depends as much on communication as on surgical expertise. Structured counseling pathways, such as the model presented in Figure 6, help integrate medical explanation with parental values. From clinical experience, carefully reviewing imaging together with families often clarifies understanding more effectively than abstract reassurance alone.

### FIF as a window into human development

Beyond its clinical implications, FIF functions as a natural experiment in asymmetric human development. It exposes how subtle deviations during monozygotic division can yield dramatic anatomical outcomes, challenging binary distinctions between normal development and pathology. In this sense, FIF is less a curiosity than a lens through which early human patterning, vascular dependence, and epigenetic divergence can be reconsidered. Its study reminds clinicians and embryologists alike that developmental processes are neither linear nor fail-safe, but contingent on timing, symmetry, and context.

## Strengths, Limitations, and Future Directions

This review brings together a highly fragmented body of literature into a coherent developmental and clinical narrative. One of its primary strengths lies in the deliberate integration of embryology, imaging, genetics, surgery, and ethical context, rather than treating FIF as an isolated surgical curiosity. By aligning historical observations with modern diagnostic pathways and molecular insights, the review attempts to restore continuity to a field that has evolved unevenly over decades. The use of structured evidence mapping, coupled with selective synthesis rather than exhaustive repetition, allows patterns to emerge despite the rarity of the condition. Importantly, the review does not treat uncertainty as a weakness to be concealed, but as a defining feature of FIF that demands careful interpretation.

At the same time, the limitations of the available evidence necessarily shape the conclusions. Nearly all published data derive from single case reports or very small series, often reported retrospectively and with variable diagnostic rigor. This constrains any attempt to quantify risk, predict outcomes, or establish firm causal mechanisms. Even where advanced imaging or genetic analysis is available, reporting remains inconsistent, and negative findings are rarely documented with the same clarity as positive ones. The absence of standardized diagnostic thresholds, long-term follow-up protocols, and uniform reporting frameworks limits comparability across studies and introduces an unavoidable degree of interpretive subjectivity.

Future progress in the field will depend less on accumulating additional isolated case reports and more on coordinated, methodologically thoughtful collaboration. International registries that capture imaging, surgical, genetic, and longitudinal outcome data could transform FIF research from anecdotal accumulation into analyzable cohorts. Advances in fetal imaging, 3D reconstruction, and molecular profiling offer opportunities to refine prenatal diagnosis and to explore developmental asymmetry at a resolution previously unattainable. Equally important is the development of conceptual frameworks that acknowledge FIF as a model of disrupted monozygotic development, rather than an outlier at the margins of pathology. Moving forward, progress will likely come not from definitive answers, but from better questions—asked across disciplines, cultures, and clinical settings—with the humility that such a rare and complex condition demands.

## Conclusions

FIF remains one of the rarest and most conceptually challenging anomalies encountered in perinatal and pediatric medicine. Its significance lies not only in its unusual anatomy, but in what it reveals about the fragility and precision of early human development. Across the assembled evidence, a consistent picture emerges of a condition rooted in asymmetric monozygotic development, shaped by timing rather than inheritance, and defined by organization rather than neoplasia. Yet despite this growing clarity, FIF resists simplification. Each case carries its own anatomical logic, clinical trajectory, and emotional weight for the families and clinicians involved.

What this review underscores is that understanding FIF requires more than diagnostic accuracy or surgical expertise alone. It demands an integrated perspective that bridges embryology, imaging, genetics, clinical judgment, and ethical awareness. Advances in prenatal detection and operative planning have undoubtedly improved outcomes, but they have also introduced new layers of decision-making that extend beyond the operating room. The rarity of FIF ensures that uncertainty will remain a constant companion, and it is within that uncertainty that careful interpretation and humility become essential clinical tools.

Ultimately, FIF should be viewed neither as a medical curiosity nor as a solved problem. It is a reminder that even well-mapped developmental pathways can diverge in unexpected ways, producing outcomes that challenge established categories. By approaching FIF with rigor, restraint, and openness to complexity, clinicians and researchers can continue to refine care while gaining deeper insight into the earliest moments of human form and variation.

## Data Availability

The authors declare that data supporting the findings of this study are available within the article.

## Abbreviations

AFP: alpha-fetoprotein
β-hCG: beta human chorionic gonadotropin
CGH: comparative genomic hybridization
CT: computed tomography
DORV: double outlet right ventricle
FIF: fetus-in-fetu
MRI: magnetic resonance imaging
PCR: polymerase chain reaction
PRISMA: Preferred Reporting Items for Systematic Reviews and Meta-Analyses
SHH: Sonic Hedgehog signaling pathway
STR: short tandem repeat
US: ultrasound
